# Sensor Fusion in Autonomous Vehicle with Traffic Surveillance Camera System: Detection, Localization, and AI Networking

**DOI:** 10.3390/s23063335

**Published:** 2023-03-22

**Authors:** Muhammad Hasanujjaman, Mostafa Zaman Chowdhury, Yeong Min Jang

**Affiliations:** 1 Department of Electrical and Electronic Engineering, Khulna University of Engineering & Technology (KUET), Khulna 9203, Bangladesh; 2 Department of Electronics Engineering, Kookmin University, Seoul 02707, Republic of Korea

**Keywords:** autonomous vehicle, AI networking, deep learning, localization, positioning, sensor fusion, traffic surveillance camera

## Abstract

Complete autonomous systems such as self-driving cars to ensure the high reliability and safety of humans need the most efficient combination of four-dimensional (4D) detection, exact localization, and artificial intelligent (AI) networking to establish a fully automated smart transportation system. At present, multiple integrated sensors such as light detection and ranging (LiDAR), radio detection and ranging (RADAR), and car cameras are frequently used for object detection and localization in the conventional autonomous transportation system. Moreover, the global positioning system (GPS) is used for the positioning of autonomous vehicles (AV). These individual systems’ detection, localization, and positioning efficiency are insufficient for AV systems. In addition, they do not have any reliable networking system for self-driving cars carrying us and goods on the road. Although the sensor fusion technology of car sensors came up with good efficiency for detection and location, the proposed convolutional neural networking approach will assist to achieve a higher accuracy of 4D detection, precise localization, and real-time positioning. Moreover, this work will establish a strong AI network for AV far monitoring and data transmission systems. The proposed networking system efficiency remains the same on under-sky highways as well in various tunnel roads where GPS does not work properly. For the first time, modified traffic surveillance cameras have been exploited in this conceptual paper as an external image source for AV and anchor sensing nodes to complete AI networking transportation systems. This work approaches a model that solves AVs’ fundamental detection, localization, positioning, and networking challenges with advanced image processing, sensor fusion, feathers matching, and AI networking technology. This paper also provides an experienced AI driver concept for a smart transportation system with deep learning technology.

## 1. Introduction

Global giant autonomous self-driving tech companies and investors such as Tesla, Waymo, Apple, Kia–Hyundai, Ford, Audi, and Huawei are competing to develop more reliable, efficient, safe, and user-friendly autonomous vehicle (AV) smart transportation systems, not only for competitive technological development demand but to also have an extensive safety issue of valuing life and wealth. According to the World Health Organization (WHO) report, yearly approximately 1.35 million [1] people are killed around the world in crashes involving cars, buses, trucks, motorcycles, bicycles, or pedestrians, and estimates that road injuries will cost the world economy USD 1.8 trillion [2] in 2015–2030. Between 94% and 96% of all motor vehicle accidents are caused by different types of human errors, found by the National Highway Transportation Safety Administration (NHTSA) [3]. To ensure human safety and comfort, researchers are trying to implement a fully automated transportation system in which errors or faults will be turned to zero.

In January 2009, Google started self-driving car technology development at the Google X lab and after long sensor efficiency improvement research, in September 2015 Google prefaced the world’s first driverless car where the car successfully rides a blind gentleman on public roads under the project Chauffeur, that was renamed Waymo in December 2016 [4]. Tesla has begun an autopilot project in 2013 and after a couple of modifications, in September 2020, Tesla reintroduced an enhanced autopilot capable of highway travel, parking, and summoning, including navigation on city roads [5]. Other autonomous self-driving car companies mentioned before are also improving their technology day by day to achieve a competitive full automation system that can provide the most beneficial experience for human safety, security, comfort, and smart transportation systems. Although nowadays the success rate for autonomous self-driving car rides during testing periods on public roads is higher than a human-driving car, it is not sufficient yet to operate full automation and causes several errors, faults, and accident records [6,7,8]. A highly sensible and error-free self-driving car is mandatory to establish reliability among people to use AVs. Light detection and ranging (LiDAR), radio detection and ranging (RADAR), and car cameras are the most used sensors in AV technologies for the detection, localization, and ranging of objects [9,10,11,12,13,14].

To ensure exact localization and real-time positioning, AVs need a more reliable and efficient four-dimensional (4D), such as height, width, length, and position, detection system at any time that helps to make errorless decisions for self-driving cars. Remote control or monitoring is also a major issue for AV performed by a global positioning system (GPS) whose accuracy and communication capabilities are not sufficient because it does not work equally in all weather conditions and situations. Some millimeters or centimeters of range accuracy is needed for AVs in a smart transportation system, where GPS only provides 3.0 m range accuracy [15].

AVs’ perception systems [16] depend on the internal sensing and processing unit of the vehicle sensors such as camera, RADAR, LiDAR, and ultrasonic sensors. This type of sensing system of AVs is called single vehicle intelligence (SVI) in intelligent transportation systems (ITS). In the SVI system, the AV measures the object vision data by cameras, the relative velocity of the object or obstacle by RADAR sensors, the environment mapping by LiDAR sensor, and ultrasonic sensors are used for parking assistance with very short-range accurate distance detection. AVs with SVI can drive autonomously with the help of sensor detection but are unable to build a node-to-node networking system because the SVI is a unidirectional communication system where the vehicles can sense the driving environment to drive spontaneously.

On the other hand, connected and AVs (CAVs) are operated by the connected vehicle intelligence (CVI) system in ITS [17]. The vehicle-to-everything (V2X) communication system is used in CVI for CAVs driving assistance. V2X communication is a combinational form of vehicle-to-vehicle (V2V), vehicle-to-person (V2P), vehicle-to-infrastructure (V2I), and vehicle-to-network (V2N). The CVI system for CAV, in general, can build a node-to-node wireless network where the central node (vehicle for V2I and V2P) or principal nodes (for V2V) in the systems’ communication range can receive and exchange (for V2N) data packs. In the beginning, dedicated short-range communication (DSRC) was used for vehicular communication. The communication range of DSRC is about 300 m. To develop an advanced and secured CAV system, different protocols are developed, such as IEEE 802.11p in Mach 2012. For effective and more reliable V2X communication, long-term evolution V2X (LTE-V2X) and new radio V2X (NR-V2X) are developed with Rel-14 to Rel-17 between 2017 to 2021 under the 3^rd^ generation partnership project (3GPP) [18]. The features of Avs and CAVs are summarized and presented in Table 1.

Depending on AVs’ nonlinear characteristics and parameter uncertainty, researchers in recent studies proposed some novel kinematic model-based and robust fusion methods for localization and state estimation (velocity and attitude) to ensure high accuracy and reliability by integrating different sensing and measuring units such as a global navigation satellite system (GNSS), camera, LiDAR simultaneous localization and mapping (LiDAR-SLAM), and inertial measurement unit (IMU) [19]. The sideslip angle estimation and measurement under severe conditions are one of the challenging sections of AV research in ITS where the researchers are proposed different approaches and models such as automated vehicle sideslip angle estimation considering signal measurement characteristics [20], autonomous vehicle kinematics and dynamics synthesis for sideslip angle estimation based on the consensus Kalman filter [21], vision-aided intelligent vehicle sideslip angle estimation based on a dynamic model [22], and IMU-based automated vehicle body sideslip angle and attitude estimation aided by GNSS using parallel adaptive Kalman filters [23,24,25]. The main challenges of those types of integrated fusion are high latency, measurement delay, and less reliability for long-distance communication in various driving conditions.

**Figure 1:**
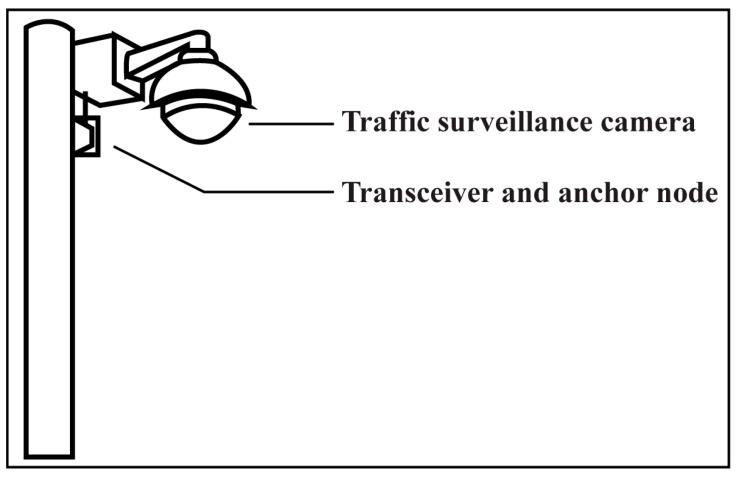
Traffic surveillance road camera as an external image source and anchor node for exact detection and AI networking system.

A new approach has been provided in this conceptual paper, fusion with a surveillance camera detection system (FSCDS), which is a 4D sensing and networking system. FSCDS can provide exact positioning and AI networking for smart transportation systems. The proposed model provides preconceptions about detecting target ground conditions that improve overall detection efficiency. It also helps in real-time monitoring, data collection, and data processing for machine learning (ML). The proposed networking system has an effective communication capability on both highways, underwater, and tunnel roads where GPS working efficiency is limited. Although AVs’ sensors can create point cloud three-dimensional (3D) modeling for object detection, the proposed detection system is more efficient and accurate due to the integration of multi-sensor systems. A traffic surveillance camera system is used as an anchor node [26] as well as an external image source shown in Figure 1 and its infrastructure after some technical modification for maintaining AI networking and communication between the AV to the base station. Experienced AI drivers (EAID) will be the next-generation AV driver with the revolution of ML, deep learning (DL), and data science technologies.

Vision sensors [27,28] are highly effective for resolution information as well as for DL, and LiDAR has exceptional mapping capability, but most of them are not based on DL. For a perfect ML and DL-based model, the system needs a very large number of data sensed by the sensors. For LiDAR sensors, 3D point cloud [29] substantiating accuracy is high [30]. In harsh and extreme weather conditions such as glare, snow, mist, rain, haze, and fog, all sensor sensing capabilities decrease exponentially. Designing an automotive system for self-driving cars that can operate perfectly in all-weather conditions is a big challenge for automation researchers. A terahertz [31] 6G (sixth generation) wireless communication system will also contribute to achieving such a system for AVs. Booming DL technology [32,33] helps to think about what the next generation AV of smart transportation systems will be. AVs are driving millions of miles and collecting data that are the primary data source for ML to train the systems and day-by-day will be capable of solving new untrained problems with the DL approach.

**Figure 2:**
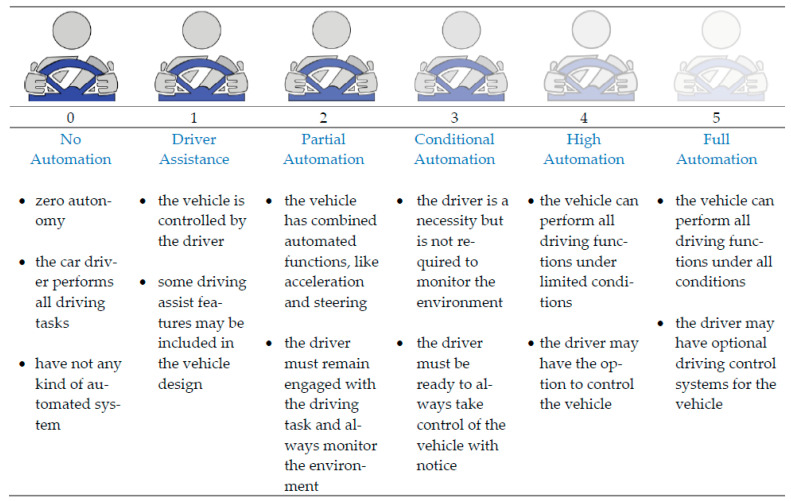
No automation to full automation levels and conditions of road vehicles where the zero level has no automation and the fifth level has a full automation system.

A fully automated system (self-driving car) design is not only a complicated task but also has major responsibility issues. In the six levels (0 to 5th) of automation shown in Figure 2, the zero level has no automation and the 5th level has full automation [34]. For the 5th level system, the vehicle can perform all driving functions under all conditions. If any fault, error, or accident occurs, then responsibility and liability will fully go into the system.

The contributions of this paper can be summarized as follows:

- Traffic surveillance camera systems are introduced for the first time with AV fusion technologies.
- For self-driving cars’ autonomous driving, 4D detection, exact localization, and AI networking accuracy improvement methodologies are shown.
- Exact localization procedure mathematical affectation is figured for joint road vehicles’ geographical positioning with the multi-anchor node positioning system.
- Deep learning-based AV driving systems and FSCDS technologies are proposed for EAID.

The rest of the paper is organized as follows. Section 2 provides a detailed overview of the related studies with problem estimations and sensor fusion technology in AVs. The proposed detection, localization, and AI networking approaches of this paper are discussed in Section 3. Qualitative detection improvement, networking performance, and finding results are presented in Section 4. The conclusion with additional thoughts and further research directions on Avs are discussed in Section 5. 

## 2. State-of-the-Art Related Works and Problems Estimation

For proper driving assistance generally, three types of sensors are used in AVs; they are camera, LiDAR, and RADAR. Laser beam reflection technology is used in LiDAR to observe the surroundings of AVs. Car cameras take video (images) and detect the object by applying advanced image processing (AIP) techniques and the Doppler properties of electromagnetic waves are used in RADAR systems to detect the relative velocity and position of targets or obstacles.

### 2.1. The Summarized Contributions Compared to Related Works

Generally, the sensor fusion technology of the camera, RADAR, and LiDAR is used in AVs for object detection, classification, and localization. For the first time, the traffic surveillance camera system is used in this work with the sensor fusion system for the 4D detection of the target whose detection and object classification accuracy are much better than the existing system because of having the actual length, width, and height of the object. Anchor node and AI networking systems are installed with the traffic surveillance camera system for exact localization and effective AI communications with AVs, where the existing GPS communication accuracy is not enough for error-free localization and communication. The mathematical affectation of the exact localization procedure is configured for joint road vehicles’ geographical positioning with the multi-anchor node positioning system. This work also provides a DL-based experienced AI driver concept for a smart transportation system with CNN technology. A comparison of the recent related studies is presented in Table 2.

Although the existing individual sensing and sensor fusion technologies of car sensors came up with good efficiency for detection and location, the proposed approaches will assist in achieving a higher accuracy of 4D detection, precise localization, and real-time positioning. Moreover, the proposed system will establish strong AI networking for AV far monitoring and data transmission systems.

### 2.2. Sensors in AV for Object Detection

**Figure 3:**
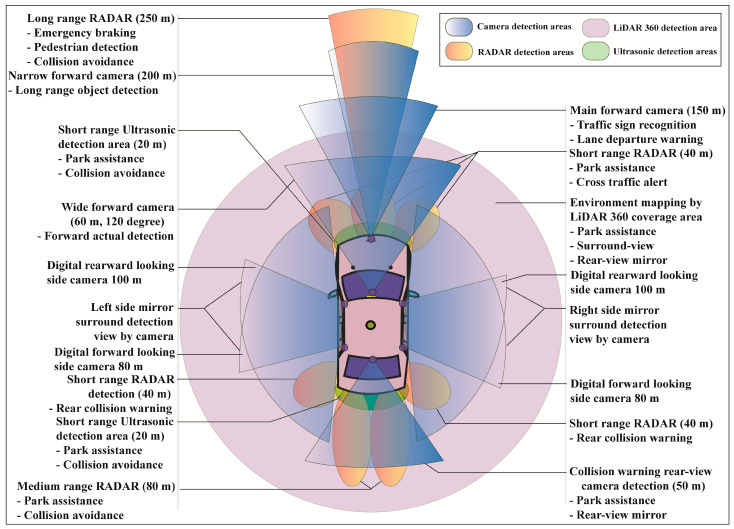
Surround sensing of an autonomous vehicle with different types of cameras, RADARs, LiDAR, and ultrasonic sensors.

Self-driving AVs are fully dependent on the sensing system of car cameras, LiDAR, and RADARs. With the combination of all sensed output data from the sensors, called sensor fusion, the AV decides whether it drives, brakes, or turns left–right, and so on. Sensing accuracy is the most crucial for self-driving cars to make error-free driving decisions. Figure 3 presents the area of an AV’s surround monitoring by car sensors for autonomous driving performance.

Generally, eight sets of camera imaging systems are used in AVs to perform different detection sensing such as one set of narrow forward cameras, one set of main forward cameras, one set of wide forward cameras, four sets of side mirror cameras, and one set of rear-view back side cameras. A narrow forward camera with 200 m capture capability is used for long-range front object detection. The main forward camera with 150 m capture capability is used for traffic sign recognition and lane departure warning. A 120-degree wide forward camera with 60 m detection capability is used for forward actual detection. Four sets of mirror cameras are used for side detection in which the first two cameras each with 100 m capture capability are used for rearward looking and another two cameras each with 80 m capture capability are used for forward monitoring. The collision warning rear-view camera with 50 m detection capability is used in an AV’s backside for parking assistance and rear-view mirror. 

Three types of different range RADARs such as low range, medium range, and high range RADARs are used for the calculation of an object’s or obstacle’s actual localization, positioning, and relative velocity. A long-range RADAR with 250 m detection capability is used for emergency braking, pedestrian detection, and collision avoidance. Three sets of short-range RADARs, each with 40 m front-side detection capability, are used for cross-traffic alert and parking assistance. Two sets of medium-range RADARs, each with 80 m backside detection capability are used for collision avoidance and parking assistance. Another two sets of medium-range RADARs, each with 40 m rear detection capability, are used for rear collision warning. Some short-range with 20 m detection capacity ultrasonic passive sensors are also used for collision avoidance and parking assistance. 

The AV’s surround is mapped by LiDAR 360, used for the surround-view, parking assistance, and rear-view mirror. In our proposed sensor fusion algorithm, the main three types of AV sensors such as camera, RADAR, and LiDAR are considered. RADAR performances for distance and relative velocity measurements are far better than the ultrasonic sensor. The AV has several cameras for the surrounding view, traffic sign recognition, lane departure warning, side mirroring, and parking assistance. LiDAR is basically used for environmental monitoring and mapping. RADARs provide cross-traffic alerts, pedestrian detection, and collision warning to avoid accidents. In AVs, short-range, medium-range, and long-range RADARs are generally used for sensor measurement purposes and ultrasonic for short-range collision avoidance as well as parking assistance.

### 2.3. Sensor Fusion Technology in AV

**Figure 4:**
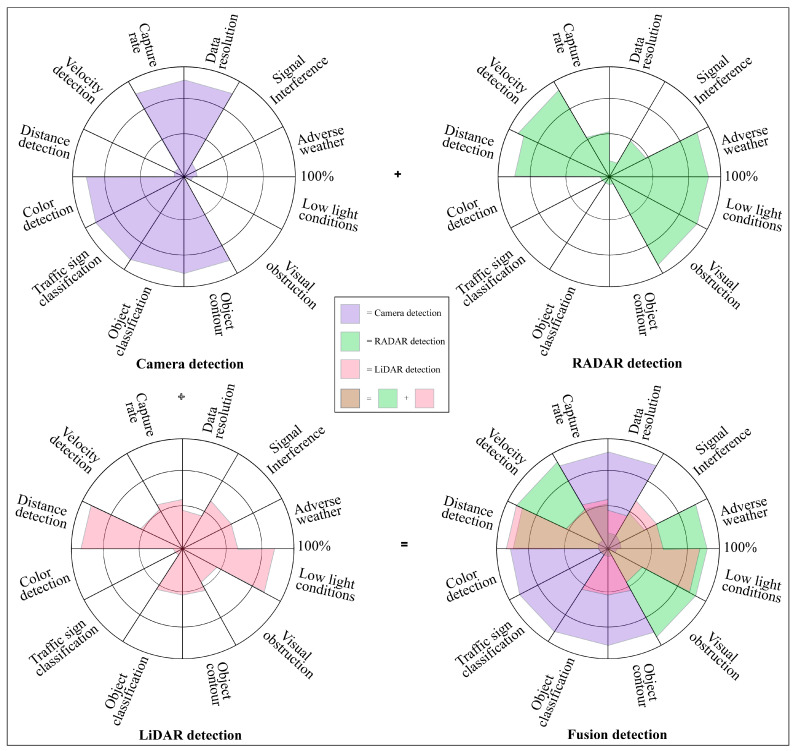
Detection capacity graphical view of AVs’ camera, RADAR, and LiDAR sensors with fusion detection.

RADAR working performances are reliable in adverse weather and low light conditions and have very impressive sensing capabilities such as relative velocity detection, visual obstruction identification, and obstacles distance measurement but its performance may be decayed by signal interference. RADAR is not good for color detection, traffic sign or object classification, object contour, capture rate, and data resolution. LiDAR also has signal interference effects, but is sovereign for 3D mapping, point cloud architecting, object distance detection, and low light working capability. The cameras’ optical sensing is signal interference-free and decent in color detection, traffic sign classification, object classification, object contouring, data resolution, and capture rate. Camera, LiDAR, RADAR, and ultrasonic sensors have individual detection advantages and limitations [49,50,51,52], which are listed in Table 3. After summarizing and graphing, Figure 4 gives an overlooked view of different sensor detection [53,54,55].

**Figure 5:**
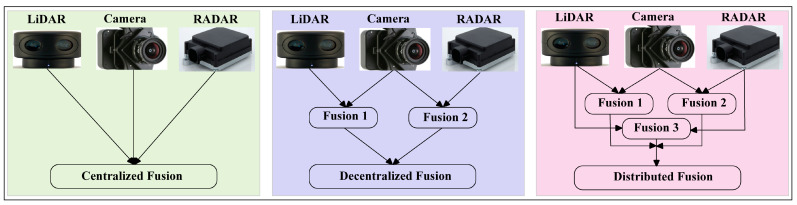
Centralized, decentralized, and distributed types of fusion technologies used for autonomous systems design. Distributed fusion technology has been used in the proposed system.

**Figure 6:**
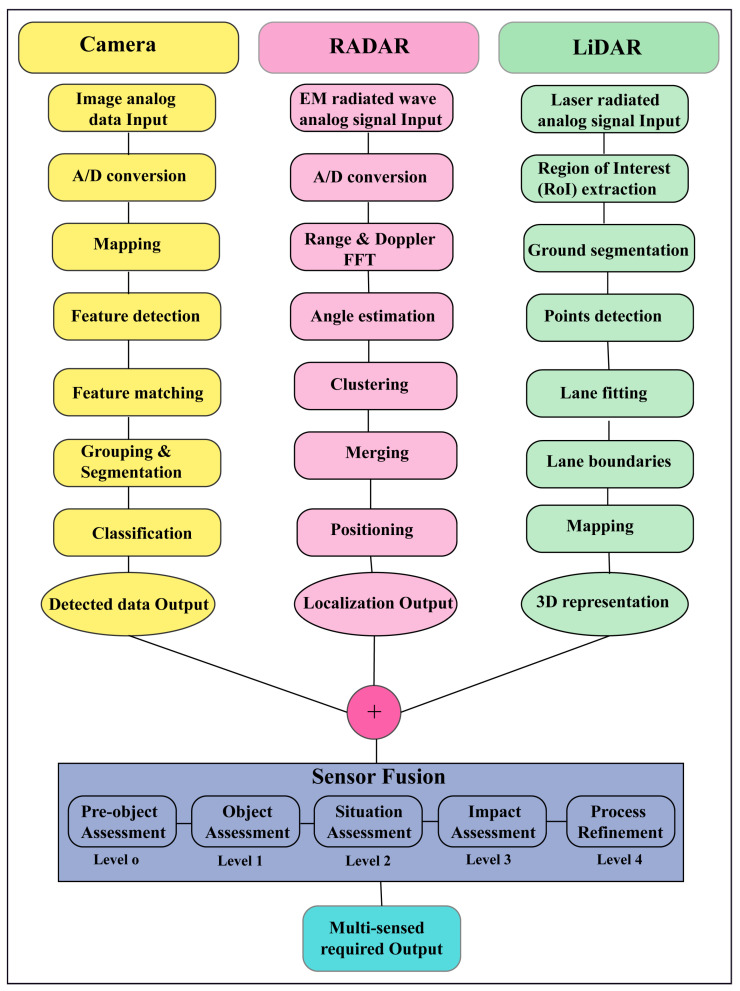
The summarized flow chart of sensor fusion technologies in AVs.

Sensor fusion also called multisensory data fusion or sensor data fusion is used to improve the specific detection task. In AVs, the primary sensors of cameras, RADAR, and LiDAR are used for object detection, localization, and classification. The distributed data fusion technology shown in Figure 5 is used in the proposed system. In five levels of data fusion technologies, wide-band and narrow-band digital signal processing and automated feature extraction are performed in the first level (level 0) fusion domain for pre-object assessment. The second level (level 1) or object assessment is the fusion domain of image and non-image fusion, hybrid target identification, unification, and variable level of fidelity. In the third and fourth levels (levels 2 and 3), situation and impact assessments are the fusion domain of the unified theory of uncertainty, the automated section of knowledge representation, and cognitive-based modulations. The fifth level (level 4) called process refinement is the fusion domain of optimization of non-commensurate sensors, the end-to-end link between inference needs and sensor control parameters, and robust measures of effectiveness (MOE) or measures of performance (MOP). The summarized flow chart of sensor fusion technologies [56,57,58,59] in AVs is shown in Figure 6.

## 3. Proposed Approaches for Detection, Localization, and AI Networking

Infront object or obstacle distance, relative velocity, surround mapping, traffic sign as well object classification, and object 3D format estimation are the most fundamental objectives of AV sensors. To establish a multi-modal and high-performance autonomous system, accurate 3D point cloud or formatting is crucial and needs the actual height, width, and length for perfect object detection [60]. The car camera can detect its front side (target back side) only, but it is difficult for the actual length measurement.

### 3.1. Detection Approach

**Figure 7:**
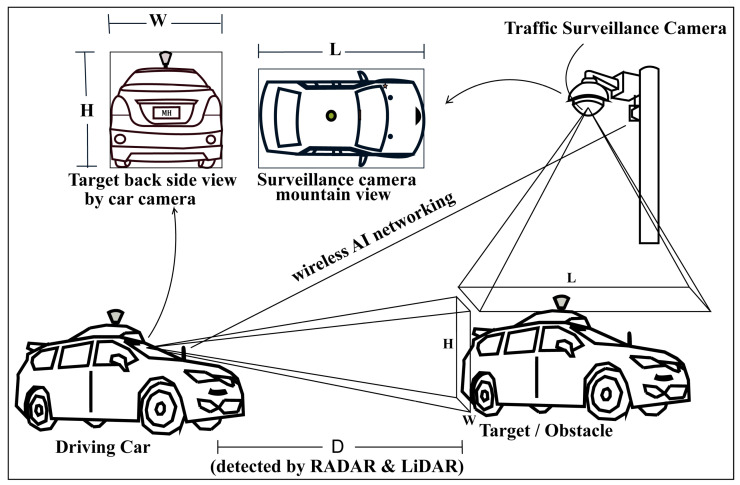
Proposed exact 4D detection and AI networking model for AV with traffic surveillance camera and car sensors.

The proposed detection approach established a hybrid system to obtain the actual 4D formations of targets or obstacles where the actual height and width measurements are received by the car camera and the actual length measurement is received by the surveillance camera birds’ eye or mountain view, shown in Figure 7. Although in the conventional system the length is calculated by LiDAR 3D for obtaining the 3D bounding box, the proposed model’s 3D formation accuracy will be more accurate because of having an actual length received from the surveillance camera system. Moreover, researchers are working to replace LiDAR [61] with advanced RADAR systems as well as multi-sensed 3D camera imaging with AIP technologies because of some commercial use limitations of LiDAR such as its high expense, high signal interference and noise, and the problem of having rotating parts. “Will have or have not LiDAR”, the detection by surveillance camera systems, will be supportive for self-driving AVs in all conditions.

Now, the resulting of the Maximum Heading Similarity (MHS) [62] metrics for sensor fusion are expressed as:

(1) $$MHS_{f_{1}}=\sum_{j=1}^{n}\max\;s(\tilde{r})\;$$

In (1), $s(\tilde{r})$ is the estimated fusion value of the AV sensors. The Average Detection Precision (ADP), $s(\tilde{r})$ [63], for the car sensors is expressed as:

(2) $$s(\tilde{r})=\hat{p}(c_{c,i})\cup \hat{p}(l_{c,i})\cup \hat{p}(r_{c,i})$$

In (2), $\hat{p}(c_{c,i})$, $\hat{p}(l_{c,i})$, and $\hat{p}(r_{c,i})$ are the individual AV detection precision of the car camera, LiDAR, and RADAR, respectively.

If the detection precision of the car sensor fusion and traffic surveillance camera are $\hat{p}(f_{c})$, and $\hat{p}(c_{tsc,i})$, respectively, then the overall ADP of fusion with the surveillance camera system is expressed as:

(3) $$s(\tilde{k})=\hat{p}(f_{c})\cup \hat{p}(c_{tsc,i})$$

For the overall fusion with surveillance camera images in (3), the MHS metrics can be expressed as:

(4) $$MHS_{f_{2}}=\sum_{j=1}^{n}\max\;s(\tilde{k})$$

**Figure 8:**
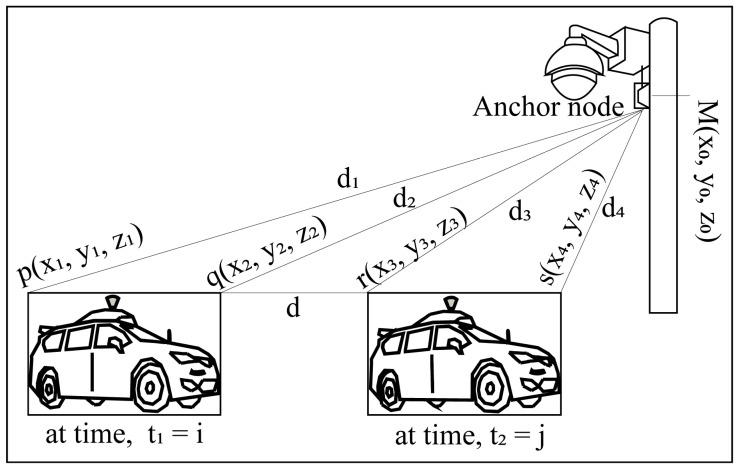
Proposed real-time positioning, localizing, and monitoring system of AVs by anchor node monitoring system.

In (4), $MHS_{f_{2}}$ is the overall fusion detection upliftment. The anchor nodes installed with traffic surveillance cameras provide real-time 4D localization and positioning information shown in Figure 8.

**Figure 9:**
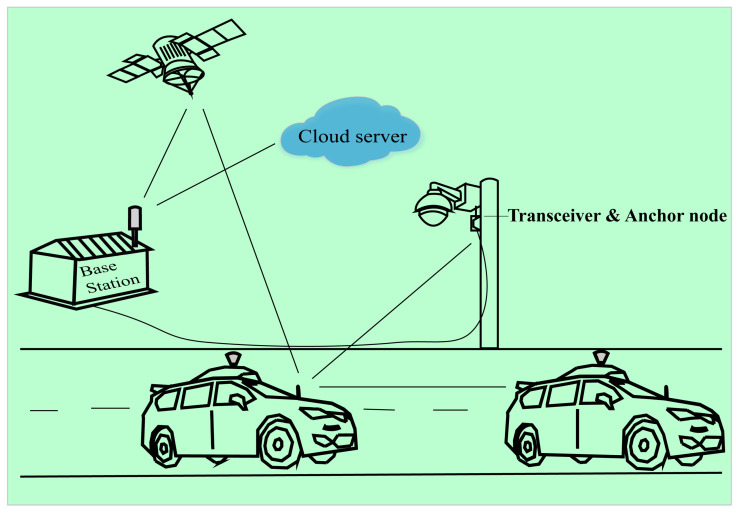
Proposed AI multi-networking technology for AV with modified traffic surveillance camera system.

AI networking is one of the crucial requirements in advanced AV technology for far monitoring and data communication. The proposed model provides real-time data transmission and communication concepts for an effective AV system where the vehicles, surveillance camera transceiver, base station, cloud internet, and satellite are connected for fruitful communication. Because of having multi-networking systems, this model works effectively for data communication and real-time positioning in tunnel roads where GPS does not work properly. The wireless AI networking model between AVs, satellites, base stations, and cloud internet monitoring is shown in Figure 9.

### 3.2. Localization Approach

**Figure 10:**
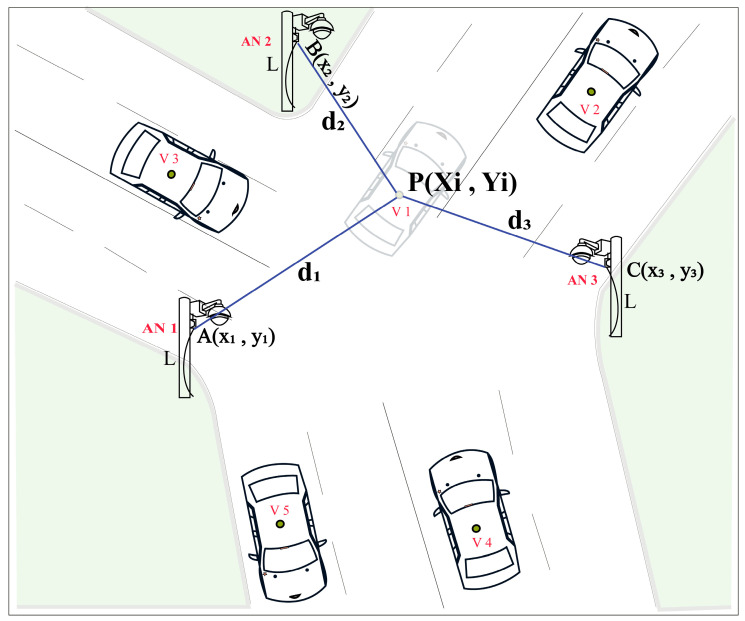
Proposed localization model of AV’s positioning by multi-anchor nodes traffic surveillance system.

The AVs’ localization calculation of the joint road position is shown in Figure 10 where $A(x_{1},y_{1})$, $B(x_{2},y_{2})$, and $C(x_{3},y_{3})$ are three anchor nodes and their distances from the unknown blind node $P(X_{i},Y_{i})$ are $d_{1}$, $d_{2}$, and $d_{3}$, respectively. The general relations among the A, B, C, and P points can be expressed as:

(5) $${\left(x_{n}-X_{i}\right)}^{2}+{\left(y_{n}-Y_{i}\right)}^{2}=d_{n}{}^{2};\;n=1,\;2,\;3\;$$

To solve those three sets in (5) of linear equations and to remove the quadratic terms $X_{i}^{2}$ and $Y_{i}^{2}$, subtracting the third equation (*n* = 3) from the two previous ones (*n* = 1, 2), resulting in two remaining equations which are:

(6) $${\left(x_{1}-X_{i}\right)}^{2}-{\left(x_{3}-X_{i}\right)}^{2}+{\left(y_{1}-Y_{i}\right)}^{2}-{\left(y_{3}-Y_{i}\right)}^{2}=d_{1}{}^{2}-d_{3}{}^{2}$$

(7) $${\left(x_{2}-X_{i}\right)}^{2}-{\left(x_{2}-X_{i}\right)}^{2}+{\left(y_{2}-Y_{i}\right)}^{2}-{\left(y_{2}-Y_{i}\right)}^{2}=d_{2}{}^{2}-d_{3}{}^{2}$$

Rearranging (6) and (7), the results can be expressed as:

(8) $$2(x_{3}-x_{1})X_{i}+2(y_{3}-y_{1})Y_{i}=(d_{1}^{2}-d_{3}^{2})-(x_{1}^{2}-x_{3}^{2})-(y_{1}^{2}-y_{3}^{2})$$

(9) $$2(x_{3}-x_{2})X_{i}+2(y_{3}-y_{2})Y_{i}=(d_{2}^{2}-d_{3}^{2})-(x_{2}^{2}-x_{3}^{2})-(y_{2}^{2}-y_{3}^{2})$$

Equations (8) and (9) can be easily rewritten as an equation of linear matrix as:

(10) $$2\left(\begin{array}{l} x_{3}-x_{1}\;y_{3}-y_{1}\\ x_{3}-x_{2}\;y_{3}-y_{2} \end{array}\right)\left(\begin{array}{l} X_{i}\\ Y_{i} \end{array}\right)=\left[\begin{array}{l} \left(d_{1}^{2}-d_{3}^{2})-(x_{1}^{2}-x_{3}^{2})-(y_{1}^{2}-y_{3}^{2}\right)\\ \left(d_{2}^{2}-d_{3}^{2})-(x_{2}^{2}-x_{3}^{2})-(y_{2}^{2}-y_{3}^{2}\right) \end{array}\right]$$

The actual position of the blind node $P(X_{i},Y_{i})$ can be easily determined by solving (10). The proposed prediction-based detection and multi-anchor positioning system improves the overall detection and reduces localization errors.

### 3.3. Deep Learning Approach

**Figure 11:**
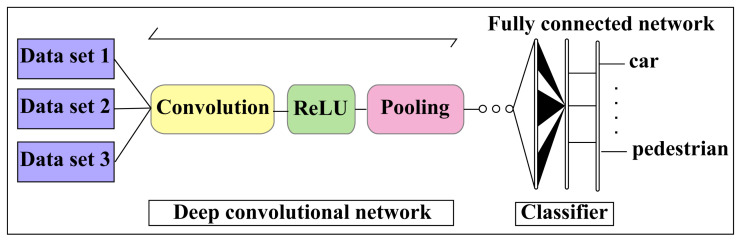
Convolutional neural networking model for DL and AI processing.

**Figure 12:**
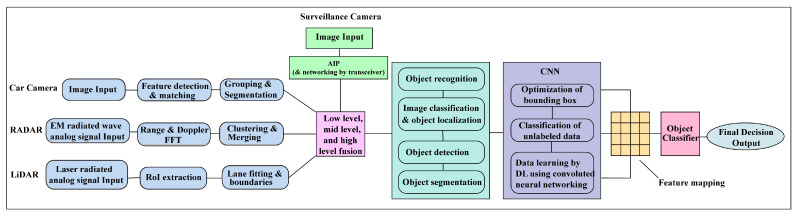
AI algorithm and CNNs architectural block diagram for surveillance camera and car sensors integrated detection system in FSCDS.

To improve the accuracy of fully autonomous driving and AI networking systems, DL technology with CNN and AI systems are applied appropriately, as shown in Figure 11. Recent research and studies have shown that DL and CNN techniques are vulnerable to adversarial sample inputs crafted to force a deep neural network (DNN) to provide adversary-selected outputs [64,65]. The combinations of fusion data and surveillance camera images estimate an accurate 4D formation of the target with a CNN, shown in Figure 12. The AV driving system will be learned during the driving period by reinforcement learning with different driving conditions and achieve smart self-decision-making capabilities in unknown conditions being experienced by AI drivers for AVs [66,67,68].

#### Dataset for Train the Model

The CARLA (CarSim) simulator has been used for different driving condition simulations in various environments. CARLA is used for advanced AV research and is popular for simulation diversity with enriched library datasets. The users can make variations independently on the demand such as driving environment, dynamic weather, number of vehicles on the road, sensor sets, and sensor range. The simulator can process the fusion data received from various sensors. For the individual sensor detection precision calculation and comparison with the proposed model, the camera, RADAR, and LiDAR sensors are used individually. To obtain fusion and FSCDS detection precision values, combinational sensing has been used. The PythonAPI for CARLA is openly available with repository examples here, https://github.com/carla-simulator/carla/tree/master/PythonAPI (accessed on 8 January 2023). For the training of the model with a big fusion date, the unScenes [69] integrated dataset is used. The unScenes dataset is a popular large-scale dataset used for AV research collected data from entire sensors such as six cameras, five RADAR, one LiDAR, GPS, and IMU. The unScenes includes 7× more object annotations compared to the KITTI dataset.

Object detection and localization are the two major parts to gain a complete image understanding of DL. In three AV sensors, the vision sensors are only DL-based but LiDAR and RADAR are not properly yet. Fast and faster regions with CNN (R-CNN) are generally used for object region detection and localization. The ImageNet dataset, Astyx Dataset HiRes2019 dataset, and Berkeley DeepDrive dataset are used for the camera, RADAR, and LiDAR detection measurements to calculate the detection precision accuracy of the proposed system. From those datasets, of the huge collection, only six objects have been chosen such as cars, bicycles, motorcycles, buses, trucks, and pedestrians. The fusion ADP and FSCDS ADP values are calculated by MHS metrics (Equations (1)–(4)) from the value calculation (for the camera, RADAR, and LiDAR) with individual datasets and the elimination process with the assumption values. The ImageNet project, a large visual database, is designed for the software research of visual object recognition where about 14 million images have been hand-annotated to indicate what objects are pictured, and one million of the image bounding boxes are also provided. Astyx Dataset HiRes2019 is an automotive RADAR-centric dataset for DL-based 3D object detection whose size is more than 350 MB and consists of 546 frames. The Berkeley DeepDrive dataset is comprised of more than 100 K video sequences with diverse kinds of annotations including image-level tagging, object bounding boxes, drivable areas, lane markings, and full-frame instance segmentation. The Berkeley DeepDrive dataset possesses geographic, environmental, and weather diversity, which is very useful for autonomous training models so that they are less likely to be surprised by new operating conditions [70].

## 4. Experimental Results with Qualitative Detection and Networking Performance Analysis of the Proposed Systems

**Figure 13:**
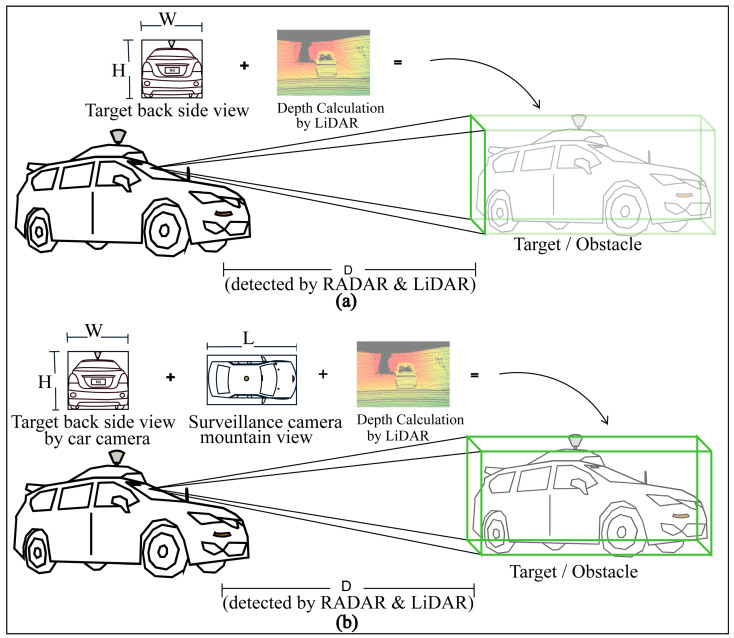
The 3D detection performance analysis: (**a**) 3D approximate partial detections by car sensors, and (**b**) 3D detection improvement with car sensor and surveillance camera view.

**Figure 14:**
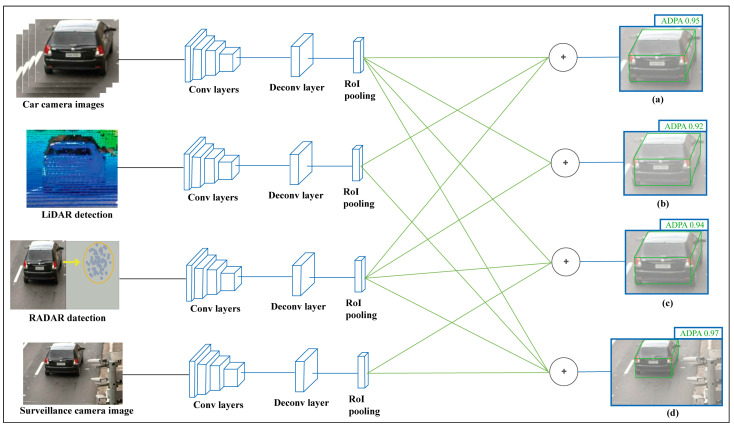
Comparative ADPA distributed fusion of different sensors. (**a**) Car camera, LiDAR, and RADAR detection, (**b**) car camera and advanced RADAR detection, (**c**) car camera, RADAR, and surveillance camera detection, and (**d**) FSCDS detection.

By using traffic surveillance cameras, the AV’s fusion detection system can obtain a clear bird’s eye view of targets or obstacles already shown in Figure 7, where there are wireless AI networking systems between the AV and traffic surveillance camera systems and can easily measure the actual length or shape of that target. A comparison of the exact detection capability is shown in Figure 13, where the 3D bounding box formation accuracy is much better in FSCDS for having exact detection data. The 2D image input from the car camera sensor can measure the obstacle behind the front car clearly and the approximate shape is calculated by using the depth calculation of the LiDAR input, which is not properly efficient for object 3D detection and point cloud mapping. A comparative average detection precision accuracy (ADPA) distributed fusion of different sensors is shown in Figure 14, whereby the combination of the surveillance camera image, car camera image, and LiDAR provides the most reliable detection and tracking performance. Table 4 provides the application and performance analysis of the proposed approach. 

**Figure 15:**
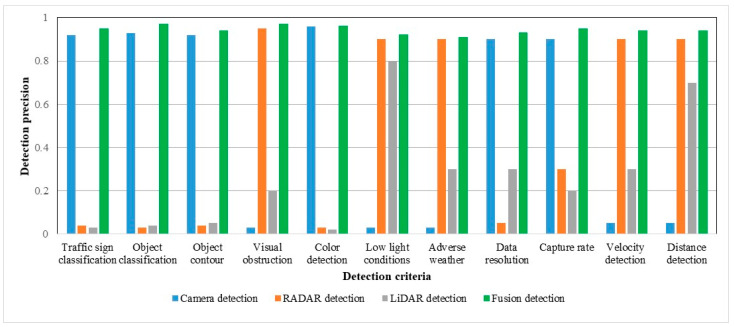
AV sensors fusion detection accuracy estimation with respect to car sensors.

**Figure 16:**
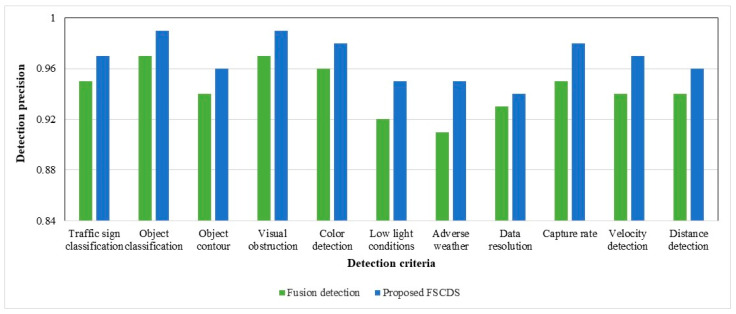
Detection estimation accuracy comparison of AV sensor fusion vs. proposed FSCDS approach.

**Figure 17:**
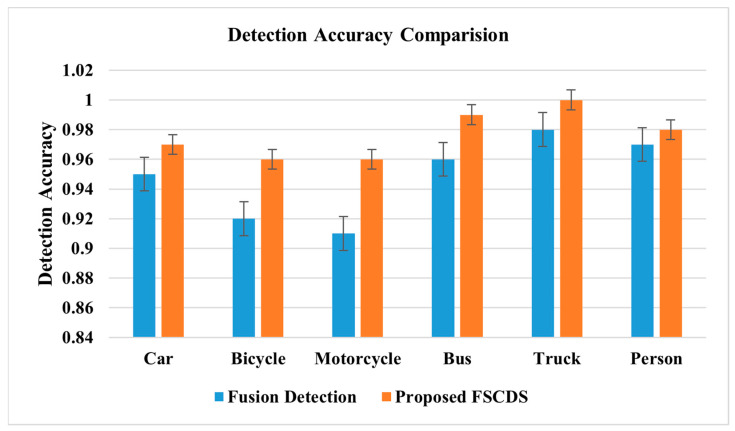
Detection accuracy comparison of fusion detection and FSCDS.

**Figure 18:**
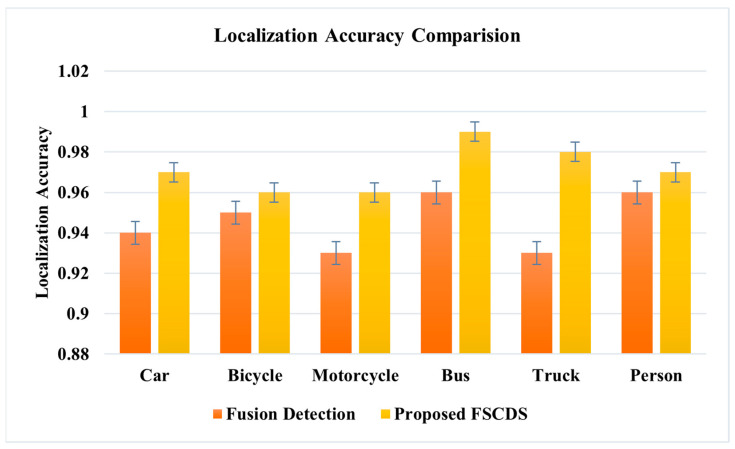
Localization accuracy comparison of fusion detection and FSCDS.

The individual approximate detection capability and a combination of sensor fusion detection are represented in Figure 15, where the detection probable assumption of sensors is between zero and one. The comparative diagram of car sensor fusion and overall fusion with surveillance camera detections is shown in Figure 16, where the proposed FSCDS’s overall detection capacity is much better than only car sensor fusion detection. In FSCDS, the surveillance camera provides extra information about obstacles, targets, and road conditions with an AI networking system that improves the overall detection capability of the fusion output. The comparison of the detection accuracy of fusion and FSCDS is shown in Figure 17, where the FSCDS detection performance is much better than the fusion value of car sensors. Figure 18 shows the localization accuracy comparison of fusion detection and FSCDS.

## 5. Conclusions

Advanced driver assistance systems for a reliable AV are mandatory in a smart transportation system which needs to have a multi-sensing, AI networking, quick and correct decision making, and intelligent operating capability under all situations and conditions. Smart detection, more accurate positioning, AI networking, remote monitoring, control, and driving in harsh weather are the maximal significant challenges for self-driving automated systems in which the proposed 4D detection and AI networking system will be capable of solving those limitations. The proposed system’s localization, positioning, 3D point cloud, and intelligent networking are much better than conventional systems because of having smart detection and AI networking which also assist to operate simultaneously in different conditions such as driving in harsh weather and tunnel road AV driving.

The EAID model has been proposed to design all AVs under a deep learning-based model because thousands of new driving decisions and conditions will be alive during driving periods and the system will be learned by ML technologies. It is also capable of taking efficient and quick decisions in an unknown situation. Although at present LiDAR has a significant role in AVs surround mapping and detection, this sensor is so expensive, has a rotating component, and noise is affected by signal interference. It is hoped that researchers will soon be able to replace LiDAR with inexpensive and efficient advanced image (optical, thermal, and so on) processing and smart AI networking technologies. AV users will feel safer, secure, and comfortable with EAID.

Further work can be carried out in the following area: The availability of data is crucial for AVs’ exact localization and networking, hence the robust algorithm will be modeled for harsh weather and adverse driving conditions; the proposed model is affluent to the wireless ITS so the security model for wireless networking will be developed for a reliable communication system and avoid malfunctioning; for EAID development, the large amount of fusion dataset will be collected for DL purpose from the real driving environment for training the AV; the effectiveness and robustness of the proposed concept will be verified by real vehicle tests in various driving environments.

## Figures and Tables

**Table 1:** The summarized features of AVs and CAVs.

Feature	AVs	CAVs
Intelligence	SVI	CVI
Networking	Sensor’s network	Wireless communication network
Range	Approximately 250 m	300 m (DSRC) to 600 m (NR-V2X)
Communication	Object sense by the sensors	Node-to-node communication
Reliability	Reliable (Not exactly defined)	95% (LTE-V2X), 99.999% (NR-V2X)
Latency	No deterministic delay	Less than 3 ms (LTE-V2X)
Direction	Unidirectional	Multidirectional
Data rate	N/A	>30 Mbps

**Table 2:** A comparison with the recent related studies.

Year	Paper	AV Applications	Sensors
2016	Schlosser et al. [35]	Pedestrian detection	Vision and LiDAR
2016	Wagner et al. [36]	Pedestrian detection	Vision and Infrared
2017	Du et al. [37]	Vehicle detection, lane detection	Vision and RADAR
2018	Melotti et al. [38]	Pedestrian detection	Vision and LiDAR
2018	Hou et al. [39]	Pedestrian detection	Vision and Infrared
2018	Gu et al. [40]	Road detection	Vision and LiDAR
2018	Hecht et al. [10]	Road detection	LiDAR
2018	Manjunath et al. [14]	Object detection	RADAR
2019	Shopovska et al. [41]	Pedestrian detection	Vision and Infrared
2019	Caltagirone et al. [42]	Road detection	Vision and LiDAR
2019	Zhang et al. [43]	Road detection	Vision and Polarization camera
2019	Crouch et al. [9]	Velocity detection	LiDAR and RADAR
2020	Minto et al. [44]	Object detection	RADAR
2021	Chen et al. [12]	Object detection	Vision and LiDAR
2022	Zhang et al. [45]	Object detection	Vision
2022	Ranyal et al. [46]	Road detection	Vision
2022	Ghandorh et al. [47]	Road detection	Vision
2023	Wang et al. [48]	Vehicle detection, lane detection	RADAR
This paper		All obstacles detection, localization, and AI networking	Sensor fusion with traffic surveillance camera

**Table 3:** Pros and cons of camera, LiDAR, RADAR, and ultrasonic detection technologies in AV systems.

Sensors	Pros	Cons
Camera	High-speed imaging Passive sensor Best for recognition No need for high power Inexpensive Infrared or thermal availability Interference-free High sensing resolution AI and deep learning research are very advanced	Light and visibility dependent Easily affected by shadow or reflections Get dirty frequently Direct 3D is not possible without any stereo
LiDAR	Direct 3D information Performed in both day and night Very high accuracy measurements High resolution At present, AI research is very advanced	Very expensive No appearance information Ineffective under rain and fog Have rotating parts Most of LiDAR is not a deep learning base yet
RADAR	Captures direct distance and velocity Inexpensive Performed both day and night Immunity to adverse weather Detect potentially long-range Reliable and proven technology Solid state	Provides very noisy output Object boundary detection is not good Limited classification capability Poor resolution Unable to detect small objects AI research just started
Ultrasonic Sensor	Has sensing capability with all material types Not affected by atmospheric dust, rain, snow, etc. Can work in all adverse conditions Provides good readings in sensing large-sized objects with hard surfaces	Air needs to travel and is easily affected by wind Highly sensitive to temperature variation and vapors Difficulties in reading from soft, curved, thin, and small objects

**Table 4:** Compressional performance analysis of traditional fusion and the proposed FSCDS.

Issues	Conventional AV Detection	Proposed FSCDS
4D detection for localization and positioning	Partially possible by AV’s sensors (camera, RADAR, and LiDAR).	Figure 7 and Figure 13 show how the proposed FSCDS perfectly detects the 4D position and exact localization for AVs.
Real-time ground preconception for smart detection assistance	Not available (GPS detection preconception assistance is not enough, even not applicable at every location and all-weather conditions).	Before starting the AV fusion sensing function, the FSCDS model provides a preconception about sensing ground that assists to prevent error sensing.
AV efficiently remote monitoring and control	The smart GPS system is used for satellite AV monitoring, but the satellite signal is attenuated by obstacles and does not work properly in harsh weather. (GPS positioning horizontal accuracy 3 m [15]).	By fitting anchor nodes with surveillance cameras and establishing wireless networking (Figure 9) between the AV and node to the base station, FSCDS establishes a strong real-time remote monitoring and control system.
Working (networking) availability in the tunnel or underwater road	Indoor working efficiency is not enough for signal attenuation.	FSCDS’s working efficiency is equally best both on highway and tunnel roads.
Point cloud 3D object modeling	Efficient [64].	FSCDS is more efficient because of the exact 4D detection (Figure 13) system.
The data rate for users	Conventional data rate [71].	In FSCDS, high-speed data communication is possible for AV users because of CNN and smart AI networking in the proposed communication system.

## Data Availability

Not applicable.

## Abbreviations

4D	Four-Dimensional
6G	Sixth Generation
ADAS	Advanced Driver Assistance Systems
ADPA	Average Detection Precision Accuracy
AI	Artificial Intelligence
AIP	Advanced Image Processing
AV	Autonomous Vehicle
CNN	Convolutional Neural Network
DL	Deep Learning
DNN	Deep Neural Network
EAID	Experienced Artificial Intelligent Driver
FSCDS	Fusion with Surveillance Camera Detection System
GPS	Global Positioning System
LiDAR	Light Detection and Ranging
MHS	Maximum Heading Similarity
ML	Machine Learning
NHTHA	National Highway Transportation Safety Administration
QoS	Quality of Services
RADAR	Radio Detection and Ranging
R-CCN	Region with Convolutional Neural Network
RoI	Region of Interest
SAE	Society of Automotive Engineers
WHO	World Health Organization
